# Epileptic seizures

**DOI:** 10.15190/d.2020.7

**Published:** 2020-06-12

**Authors:** Haleema Anwar, Qudsia Umaira Khan, Natasha Nadeem, Iqra Pervaiz, Muhammad Ali, Fatima Fayyaz Cheema

**Affiliations:** ^1^CMH Lahore Medical College & Institute of Dentistry, Lahore, Pakistan

**Keywords:** Epilepsy, focal epilepsy, postictal Todd’s paresis, EEG, gene therapy.

## Abstract

Epilepsy is a condition marked by abnormal neuronal discharges or hyperexcitability of neurons with synchronicity and is recognized as a major public health concern. The pathology is categorized into three subgroups: acquired, idiopathic, and epilepsy of genetic or developmental origin. There are approximately 1000 associated genes and the role of γ-aminobutyric acid (GABA) mediated inhibition, as well as glutamate mediated excitation, forms the basis of pathology. Epilepsy is further classified as being of focal, general or unknown onset. Genetic predisposition, comorbidities and novel biomarkers are useful for prediction. Prevalent postictal symptoms are postictal headache and migraine, postictal psychosis and delirium, postictal Todd’s paresis and postictal automatisms. Diagnostic methods include electroencephalography (EEG), computed tomography scan, magnetic resonance imaging (MRI), positron emission tomography, single photon emission computed tomography and genetic testing; EEG and MRI are the two main techniques. Clinical history and witness testimonies combined with a knowledge of seizure semiology helps in distinguishing between seizures. Clinical information and patient history do not always lead to a clear diagnosis, in which case EEG and 24-hour EEG monitoring with video recording (video-EEG/vEEG) help in seizure differentiation. Treatment includes first aid, therapeutics such as anti-epileptic drugs, surgery, ketogenic diet and gene therapy. In this review, we are focusing on summarizing published literature on epilepsy and epileptic seizures, and concisely apprise the reader of the latest cutting-edge advances and knowledge on epileptic seizures.

## SUMMARY

*1. Introduction*

*2. Pathology and etiology*

*3. Types of epileptic seizures*

- *3.1. Focal seizures*

- *3.2. Generalized seizures*

*4. Prediction and prevention*

*5. Post seizure symptoms*

*6. Diagnosis*

*7. Differential diagnosis*

*8. Treatment*

- *8.1. First aid *

- *8.2. Drug-based therapeutics *

- *8.3. Surgery *

- *8.4. Ketogenic diet *

- *8.5. Gene therapy *

*9. Conclusion*

## 1. Introduction

The World Health Organization (WHO) and its partners have recognized epilepsy as a major public health concern. Epilepsy occurs due to hyperexcitability and an imbalance between excitation and inhibition, leading to seizures^1^. According to the WHO, around fifty million people worldwide are affected by epilepsy, making it one of the most common neurological diseases globally. Epilepsy is a neurological disorder characterized by recurrent seizures caused by sudden surge in electrical activity of the brain. This is due to abnormal neuronal discharges or hyperexcitability of neurons with synchronicity. However, the frequency of these seizures varies for different people.

Epilepsy is a multifactorial neuronal disorder. Epileptic seizures are abnormal jerky or trembling movements in the body due to abnormal neuronal activity and can result in damage to the brain or other parts of the body. Even a single seizure can cause changes in neural development and can lead to behavioural and cognitive changes. Epileptic seizures have adverse clinical characteristics^2^. These seizures have a negative impact on the lives of patients especially those who have frequent reoccurrence. The epileptic seizures cause emotional, behavioural and neurological disturbances in patients. Seizures can occur in various regions of the brain and the degree of effectiveness depends upon the characteristic area, types of seizures and the area where abnormal neuronal activity is occurring^1^. Epileptic patients suffer from social stigma and discrimination; misconception and negative attitudes of society towards this disorder may prevent epileptic patients from seeking treatment and leading a confident life.

This review briefly covers the pathology and classification of epileptic seizures. It also highlights prediction and prevention, diagnosis, differential diagnosis and the various available treatments, including drugs, surgical excision, dietary therapy and gene therapy for epileptic seizures.

## 2. Pathology and etiology

Epilepsy is classified into three categories based on the etiology, named acquired, idiopathic, and epilepsy of genetic or developmental origin. Idiopathic epilepsy is without neurological signs, and its onset is in childhood. Some examples of idiopathic epilepsies are childhood absence epilepsy and juvenile myoclonic epilepsy. Acquired epilepsy is due to identifiable structural abrasions of our brain. The causes of acquired epilepsy are cerebral trauma, cerebral tumor, cerebral infection, hippocampal sclerosis, cerebrovascular disorders, cerebral immunological disorders and perinatal and infantile causes. Some examples are epilepsy caused by open head surgery, viral meningitis, meningioma, cavernous hemangioma and cerebral infarction. Cryptogenic epilepsy has an unknown etiology. Among acute and remote causes, etiology can be difficult to identify^3^. In modern studies, the term cryptogenic is discouraged because it conveys unclear implications. It is replaced with probably symptomatic, which provides clear implications^4^. Most studies reveal that 40 out of 100 cases of epilepsy have known etiology that includes ischemic stroke, infections in the central nervous system, brain injury, prolonged symptomatic seizures intracerebral hemorrhage, and neurodegenerative diseases.

**Figure 1:**
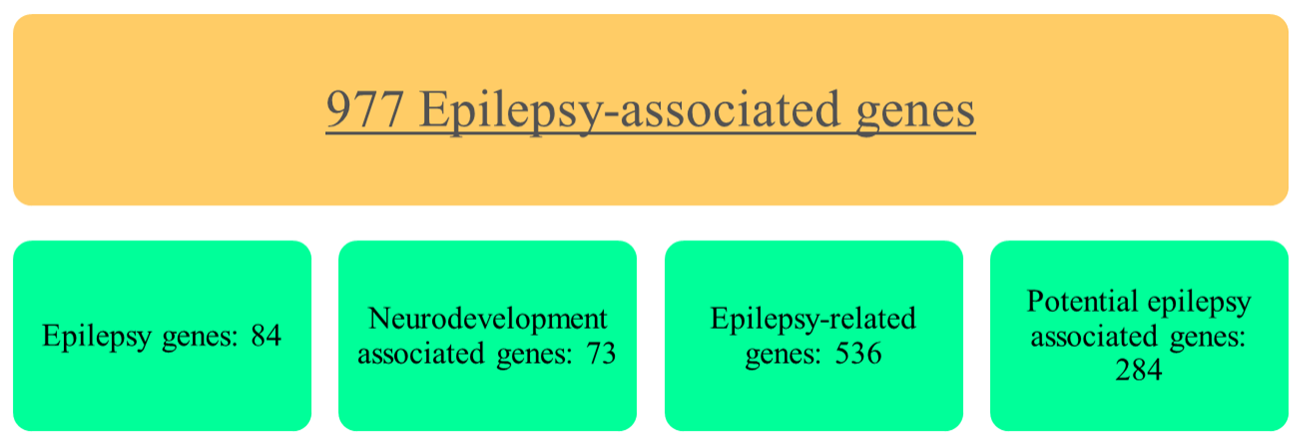
Genes associated with epilepsy (as determined by Wang J et al.^5^)

A research study published in 2016 revealed 977 epilepsy-associated genes. These were grouped into four categories, according to the phenotype. These genes are controlling the ion-channel, enzyme or enzyme regulator genes, transporter and other aspects of the cell, such as cell adhesion. 84 genes are associated with syndromes that have epilepsy as the core feature, 73 are neurodevelopment-associated genes related to brain developmental problems which can cause epilepsy, 536 epilepsy-related genes are associated with metabolic errors or other systemic abnormalities where epilepsy is not the central symptom, but rather it is one of the many clinical manifestations and 284 were potential epilepsy-associated genes that require further investigation^5^. This has been shown in Figure 1**.**

Epileptogenesis is fundamentally a biological process which leads to the appearance of the initial recurring epileptiform event, as well as to spontaneous seizures after brain insult. It involves the development and progression of epilepsy in the patient. Epileptogenesis involves biological processes, structural, as well as functional changes. Several neurotransmitters play an important role in the epileptic mechanism. The most important neurotransmitters are serotonin, dopamine, γ-aminobutyric acid (GABA), glutamate, and noradrenaline.

Two neurotransmitters are usually studied in reference to epilepsy: GABA and glutamate. In epilepsy, neuronal hyperexcitability is due to variation in GABA mediated inhibition as well as in glutamate-mediated excitation. Glutamate can depolarize the neurons, generating excitatory post-synaptic potentials. During the initiation and progression of epilepsy, specific glutamatergic molecular mechanisms occur, which includes an increase in the concentration of extracellular glutamate, upregulation of the glutamate receptors, and certain abnormalities in the glutamate transporters. These mechanisms cause hyperexcitability due to excessive glutamatergic activity.

GABA is one of the primary inhibitory neurotransmitters. It generates the presynaptic potentials through hyperpolarizing the neurons. It plays a vital role in counterbalancing the neuronal excitation, as well as in suppressing epileptiform discharges. Two GABA receptors named GABAA and GABAB are involved in the epileptogenesis^6^. Loss of GABAergic mechanisms can increase the risk of epilepsy in an individual. One of the primary transporters of GABA is GAT-1. It is accountable in re-uptake of GABA from the synapse. A mutation in the GABA transporter is also a cause of epilepsy with myoclonic, atonic seizures in many individuals^7^.

## 3. Types of epileptic seizures

International League Against Epilepsy (ILAE) proposed a classification of epilepsies and epileptic syndrome in 1989. This classification was based on symptoms which grouped the epilepsies as either generalized or focal. The classification was also made based on etiology into two groups: idiopathic epilepsies and symptomatic epilepsies. Idiopathic epilepsies were due to genetic causes and were characterized by a normal background electroencephalography (EEG) and no brain lesions. Symptomatic epilepsies, as opposed to idiopathic, were characterized by brain lesions (either focal or diffused). In 2006, a new base for categorizing epilepsies was devised by ILAE Task Force on Classification and Terminology. It included seizure type, age of onset and interictal EEG. Subcategories of epilepsy syndrome according to age of onset were neonatal period, childhood, adolescence, special epilepsy conditions, and conditions with epileptic seizures that do not require diagnosis (e.g. febrile seizures). According to the type of seizure, the subcategories were self-limiting epileptic seizures (which included generalized onset, focal onset and neonatal seizures) and status epilepticus.

The classification was further extended in 2010^1,8^. Currently, the latest revision was published in 2017. This revision was made because some important seizures were not included in previous classification, few seizures can have both generalized and focal onset and some seizures were not classified on the basis of shallow knowledge about their onset.

**Figure 2:**
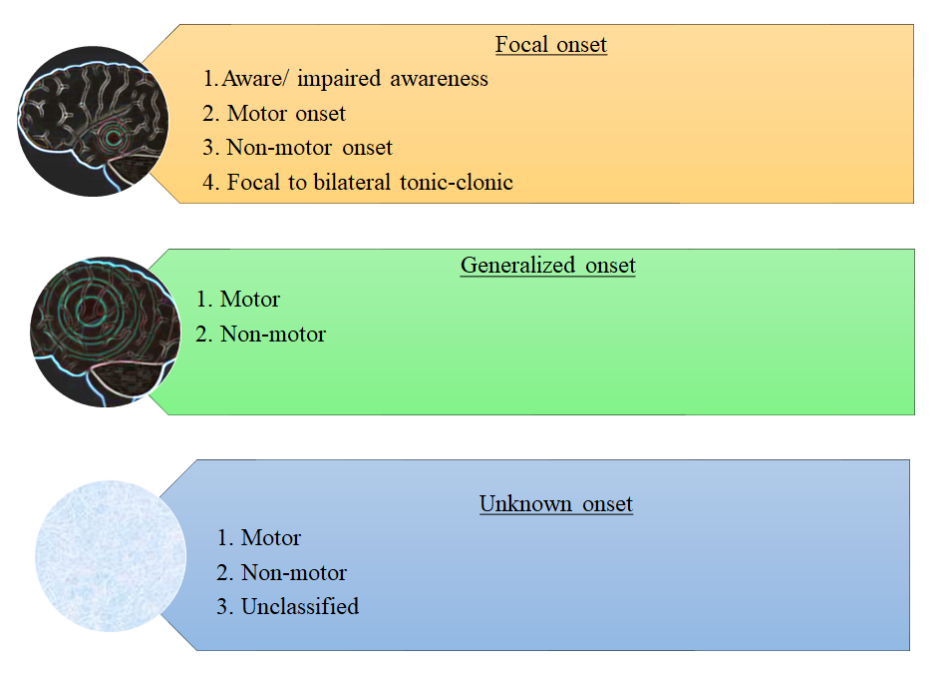
Epilepsy classification proposed by ILAE (2017)

In the 2017 revision, certain terms were added. Hyperkinetic seizures were added to focal seizure category, cognitive was used instead of ‘psychic’ (so that cognitive disabilities during seizures can be identified e.g. aphasia)^1,8^.The 2017 classification provides a much more lucid nomenclature and includes previously missing seizure types. An overview of the 2017 revised classification is shown in Figure 2.

In all these modifications to classification over the years, the terms focal and generalized epilepsies have not been disturbed. These are discussed as following.

### 3.1. Focal seizures

Focal seizures are the seizures that occur in a small localized region of the cerebral cortex or in a deeper structure of the cerebrum. A phenomenon called ‘jacksonian march’ is associated with this type of seizures. Jacksonian march is a progressive ‘march’ of muscle contraction caused by spread of neuronal discharge over the motor cortex. The muscle contraction occurs on the opposite side of the body and the direction may be from mouth region to the legs or vice versa.

In ILAE classification (according to the latest revision), focal seizures have the following major subcategories: aware, impaired awareness, motor onset, non-motor onset, focal to bilateral tonic-clonic seizures. Motor onset includes automatism, atonic, clonic, epileptic spasms, hyperkinetic, myoclonic and tonic seizures. Nonmotor onset comprises autonomic, behavior arrest, cognitive, emotional and sensory seizures.

In focal impaired awareness seizures, the person must reorient himself, whereas in focal aware seizure the person is fully aware during the seizure. This can be easily understood by a clinical scenario. A woman in her twenties experiences a seizure. During the seizure, she can hear other people talking. However, after the episode is over, she cannot recall what was being said. This is classified as focal impaired awareness. A man in his twenties has a seizure during which he is aware. He feels as if he is being flashed. This is a scenario of focal aware autonomic seizure^9^.

Focal clonic seizures can be understood by the following case: a baby boy has rhythmical jerks of the arm on one side^9^. On repositioning the jerks do not remit and EEG displays right frontal ictal rhythms. As the EEG displays a localization of the electrical discharge, therefore the seizure is focal. There is no knowledge about the awareness. Thus, awareness is not involved in the classification. As clonic seizures are a subtype of motor onset seizures, this can also be named focal motor onset clonic seizures.

### **3.2. Generalized seizures

These seizures affect both hemispheres of the brain. In the 2017 classification, they have been broadly classified into two categories: motor and non-motor. Motor seizures include tonic-clonic seizures, clonic, tonic, myoclonic, myoclonic-tonic-clonic, myotonic-atonic, atonic and epileptic spasms. Non-motor seizures include typical, atypical, myoclonic and eyelid myoclonia.

## 4. Prediction and prevention

Currently, there is no reliable non-ictal biomarker capable of tracking epileptogenesis and concurrent human acquired epilepsies with reliable accuracy and specificity^10-12^. The most relevant markers are based on the EEG, particularly pathologic high‐frequency oscillations (pHFOs) which are brief EEG events in the range of 100 to 600 Hertz. These are speculated to reflect summated action potentials in hyperexcitable neurons. Yet, to improve the scope of prediction and prevention (by means of reoccurrence or otherwise), further discussion on the genetic predisposition, associated and indicated comorbidities, as well as other novel biomarkers is considered relevant.

There is a strong genetic component to the occurrence of epilepsy, with estimated hundreds of genes contributing to the pathogenesis. Identification of a novel CHRNA4 (OMIM 118504) variant in a pedigree with benign childhood epilepsy with centrotemporal spikes (BECTS) broadens the scope for prediction of at-risk subjects by means of genetic screening^13^. Epileptogenesis is the process by which a normal brain develops epilepsy. This phase of genesis is marked by changes, mostly in neurons and glial cells, particularly in methylation of epilepsy-relevant genes HDAC11, SPP1, GAL, DRD1 and SV2C^14^. Furthermore, abnormal methylation of RASgrf1 and its subsequent downregulation has been observed in the temporal neo-cortex of epileptics^14,15^. The methylation of genes coincides with the incidence of general epilepsy, whereby it serves both as a novel target for treatment and an indicator for the likelihood of seizure occurrence.

Also, under speculation is the involvement of ion channels in genetic epilepsy where an estimated 25% of genes in epilepsy code for ion channels^16^. Hence, this furthers the claim in observed literature of the association of a hyper-excitable visuo-motor system to the development of photosensitive epilepsy. The underlying difference between photosensitive epilepsy and those without sensitivity is the decreased alpha inhibition of the visuo-motor system due to a difference in the generation of alpha oscillations by the cortical-subcortical system. Thus, the identification of the “alpha phenotype” may be useful in predicting and preventing photosensitive epileptic reoccurrences/occurrences in those that show a genetic inclination^17^.

The mouse model of investigating post-infection (malarial) acquired epilepsy has proven useful in furthering the claim of a brain-heart interaction as a biomarker bearing relevance to epileptogenesis. On a beat to beat interval the aberrant cortical discharge precedes the change in heart activity, with this deviation from the norm being identifiable 2-14 weeks before the seizures^10^.

Epilepsy-associated comorbidities (intellectual disability (ID), anxiety disorder and attention-deficit/hyperactivity disorder (ADHD), depression & autism spectrum disorders) may share common pathological mechanisms with epileptogenesis and in certain cases have been increasingly recognized as an early symptom of the developing epilepsy. However, they have yet to be investigated and quantified in their predictive value^12^.

Trigger avoidance may as well be a hallmark of preventing seizures, whereby common triggers to be avoided include sleep deprivation, alcohol consumption, physical and mental exhaustion, metabolic disturbances and missed dosage of an antiepileptic drug (in treated patients). On the topic of reflex epilepsies, prevention is based on the avoidance of relevant triggers for the subject on a case by case basis^18^. Common triggers can be external (flashing lights, hot water), internal (emotional thinking) or both. It would be advised to make informed changes in lifestyle with flickering lights, distance to screens, intensity of images and the refresh rate of television screens (a 100 Hertz television display being less liable to inducing a seizure as compared to a 50 Hertz one) in known photosensitive subjects.

Identification of biomarkers is limited by an ill-defined window of time (e.g.: EEG changes in the acute time following a traumatic insult). Genetic predispositions are unlikely to be the primary predictor, since the patients will mostly come to clinicians after onset of symptoms of seizure. A single biomarker might prove to be insufficient in predicting the epileptogenesis or a preictal period, while multiple biomarkers may be the necessary requirement.

## 5. Post seizure symptoms

Postictal state is an abnormal condition, occurring from the end of an epileptic seizure and lasting until the return to a normal neurological function.The postictal period is characterized by changes in behaviour, motor function and neuropsychological performance. The type, intensity and duration of these symptoms is variable, differing even in the same patient from seizure to seizure^19^.

A systematic review determined that postictal symptom duration ranged from 3 seconds for postictal automatisms to about 12.3 days for postictal depression and anxiety. A 2019 meta-analysis found up to 31 postictal symptoms including depression, anxiety, irritability, hypersalivation, euphoria, hypomania, sleep, coma, lethargy, fatigue, vomiting, anorexia, laughter and sighing^20^. The more prevalent postictal symptoms are postictal headache and migraine, postictal psychosis and delirium, postictal Todd’s paresis, postictal aphasia, postictal cognitive deficits (confusion and memory loss) and postictal automatisms.

Postictal migraines are the second-most frequently occurring with a mean weighted overall estimate of 16.0% of epileptic patients experiencing them. Postictal headaches and migraines occur more frequently in children than in adults and postictal migraines occur more often in women than in men (63.0% vs 33.3%)^21^. The mean duration of postictal headaches was found to be 14 hours. This symptom generally manifests within 3 hours of an epileptic seizure and can continue up to 72 hours.

A study in 2018 found that the clinical pattern of headache correlated to seizure onset and epileptic patients demonstrated different types of headaches. Its results showed that tension-type headaches occurred in 73.3% of patients who experienced postictal headaches^22^.

A systematic review included a finding in which 66.4% of patients who experienced postictal headaches experienced this symptom after every single seizure. It has been speculated seizures can result in cerebral edema which greatly increases intracranial pressure; this indicates a potential cause for postictal headaches and migraines.

Postictal delirium typically lasts for hours but may continue up to 1 to 2 days. It is often of the hypoactive type but may evolve into the hyperactive type. During delirium, patients may become agitated or aggressive resulting in postictal violence. This symptom often occurs after complex partial or generalized epileptic seizures. Postictal psychosis is associated with hallucinations, delusions, delirium and amnesia^23^.

Todd’s paralysis (loss of motor function in the regions of the body involved during the seizure) has a good lateralizing value, pointing to the contralateral hemisphere as the site of seizure onset^24^. Practically, the concept of “Todd’s paralysis” has been broadened to include manifestations other than motor function, including postictal sensory deficits that present as somatosensory, oculomotor or auditory symptoms. The inability of patients to speak in the postictal state is referred to as postictal aphasia and lateralizes to a seizure originating in the speech-dominant temporal lobe. The postictal aphasia occurs only when the epileptic activity spreads to language areas, hence, it has limited localizing value.

The length of the aphasic period is dependent on the hemisphere of seizure onset and, in a 2017 retrospective study, was found to occur with left hemisphere seizure onset or with seizures spreading from right to the left hemisphere^25^. Postictal aphasia frequently occurs following left temporal lobe epilepsy.

Postictal automatisms are not a voluntary response and may involve the mouth (lip smacking, swallowing, chewing) or vocalization (grunts, repetition of phrases). Hand automatisms are a prominent sign of focal seizures, often manifesting unilaterally. Repetitive movements include postictal nose wiping and indicate an ipsilateral focus for seizure onset^26,27^.

## 6. Diagnosis

With adequate treatment most epileptic patients can live a normal and healthy life, but some patients develop serious mental illnesses. Therefore, continuous medical assistance may be needed**. **Early diagnosis can improve the medical condition of the patients. However, even in developed countries, 10% of patients do not get appropriate treatment, whereas in low-income countries, the percentage is 75%^28^.

Several methods are used for the diagnosing epileptic seizures. These methods include, EEG, computed tomography (CT) scan, magnetic resonance imaging (MRI), positron emission tomography (PET), single photon emission computed tomography (SPECT) and genetic testing. Simple blood tests are also done as they can be a helpful tool for describing the etiology of toxic and metabolic encephalopathies^29^. Studies suggest that EEG and MRI are the two principle techniques used in the diagnosis of epileptic seizures. Additional techniques help to confirm diagnosis and can even identify false negative results.

EEG is considered to be the most effective method to diagnose epilepsy. Whenever an unusual EEG is observed, it helps in identifying whether the seizure is a focal or generalized epileptic seizure and it may also rule out the patient’s epilepsy syndrome. Thus, it can help in the prognosis of the disease and control of further seizures, allowing for better treatment choices to be made. When nothing abnormal is observed in brain imaging, surgery is opted as a treatment of choice after Video-EEG monitoring (vEEG) confirms the injured areas of the brain^30^**.**

CT scan also proves helpful, but the detection rate of the focal lesions in the patients is only 30%. When neurosurgical treatment is being opted, neuroimaging is of critical significance in the evaluation of epilepsy. With progress in MRI technology, acquisition protocols and methods, brain imaging has improved to a great extent. Structural MRI is the main neuroimaging technique which is used for the identification of an epileptogenic lesion^31^. However, there are chances of false negatives in about 15% to 30% of patients suffering from refractory focal epilepsy.

If a patient has no distinct lesions on MRI then the latest MRI hardware and techniques are used, which may show some minute abnormality. However, this should be handled with great vigilance, since there is a possibility of a false-positive result^32^**.** There are certain reasons to use MRI as a means of investigation of the disease. MRI can help in determination of the cause and evaluation of the condition before a surgery^33^.

PET and SPECT are functional imaging studies that aid in identifying the epileptogenic zone and to allow pre- surgical evaluation. In order to find the cause of some types of epilepsies, genetic testing is used. However, there are a lot of limitations, including the relatively high cost and lack of availability^34^. PET imaging and SPECT imaging are techniques used to localize the area of cortex responsible for initiation of seizures, especially in patients with focal epilepsy who have a normal MRI, patients with multiple abnormalities, or in patients with inconsistencies between MRI and EEG. Information regarding changes in the cerebral perfusion is also provided by SPECT imaging technique^35^.

## 7. Differential diagnosis

There are three different types of seizures: epileptic seizures (ES), psychogenic nonepileptic seizures (PNES) and physiological nonepileptic events^36^ (syncope, transient ischemic attacks, parasomnias, migraines with aura, paroxysmal extrapyramidal movement disorders, vestibular syndromes, drop attacks, hypoglycemia, panic attacks, paroxysmal sleep disorders).

The main misdiagnoses of epilepsy are PNES and syncope^37^. The most common misdiagnosis is PNES. PNES presents as paroxysmal signs and symptoms imitating epileptic seizures which makes it difficult to distinguish it from epileptic seizures. 10% to 15% of patients with long-term PNES turn out to additionally have epilepsy^36^.

Differential diagnosis between epileptic and nonepileptic events of psychogenic and physiological origin can be done on the basis of clinical history and witness testimonies. Clinical history combined with a knowledge of seizure semiology helps in distinguishing between seizures^38^.

History helps in identifying syncope and ruling out epileptic seizures. If the patient’s seizure happens shortly after standing up or diaphoresis occurs before the seizure, then vasovagal syncope should be considered^39^. Semiological characteristics are especially important for differentiation between ES and PNES and clinical signs. Symptoms associated with these seizures are contrasted in Table 1.

**Table 1:** Clinical features associated with epileptic and psychogenic nonepileptic seizures^36,39^

Clinical Features	Epileptic Seizures (ES)	Psychogenic Non-epileptic Seizures (PNES)
Duration	Less than 10 minutes	Longer than 10 minutes
Seizure occurrence while sleeping	Yes	No
Seizure occurrence during pseudo sleep	No	Yes
Aura	Difficult to describe	Factual description
Reactivity during attack	Restricted or no clear reaction	Defence
Asynchronous movements	No	Yes
Prolonged periods of body flaccidity	Occur	Do not occur
Tongue biting	Lateral	Apical
Head	Fixed, unilateral	Side-to-side movements
Eyes	Open	Closed
Clenching of the jaw	Occurs during tonic phase of convulsion	Does not occur during tonic phase of convulsion
Pupillary response	Not normal	Normal
Heart rate from preictal to ictal phases	Increased	Not increased
Occurrence of vocalization	At beginning of seizure	During or after seizure
Vocalization	No	Yes
Postictal headache	Yes	No
Recollection of items memorized during seizure	No	Yes

However, clinical information and patient history do not always lead to a clear diagnosis, in which case EEG and 24-hour vEEG help in seizure differentiation. A normal EEG recording cannot rule out epilepsy because some epileptic seizures, such as simple partial epileptic seizures and frontal lobe epileptic (FLE) seizures, may show scalp-negative EEG findings. Therefore the “gold standard” for differential diagnosis of epileptic seizures is a combination of video recording, EEG and electrocardiography (ECG). vEEG recordings are a reliable way of differentiating between ES, PNES and non-epileptic seizures of psychological origin. When EEG is normal prior to, during and following the epileptic episode and vEEG recordings support clinical features that are associated with PNES, then a diagnosis of PNES can be made. Similarly, dampened amplitude and slowing of the brain waves on EEG indicate a non-epileptic seizure of physiological, possibly cardiac, origin, while positive EEG activity with supported clinical history indicates an epileptic seizure^39^.

However, vEEG may not always capture the necessary events needed for differential diagnosis and may not be able to distinguish between PNES and FLE seizures. Combining vEEG with seizure semiology makes for a more accurate diagnosis.

Other measures for differential diagnosis include prolactin levels, neuropsychological testing, provocative testing and single photon emission computed tomography. Neuropsychological test scores can differentiate between ES and PNES. Standardized tests – capable of measuring intellectual functioning, attention, memory, language skills, processing speed, executive function and emotional functioning – are carried out as part of a comprehensive neuropsychological battery^40^. PNES patients have been shown to have significantly higher intellectual functioning. This method of psychometric testing is a potential tool for differential diagnosis, especially in uncertain cases of patients with non-diagnostic vEEG.

Neurophysiological assays can also be used. They can distinguish between complex partial epileptic seizures and PNES. This is due to the association of epileptic seizures with elevated serum prolactin levels. The increase depends on which limbic region was involved during the seizure. However, one drawback is that neurophysiological assays cannot differentiate between syncope and epileptic seizures, or between PNES and partial epileptic seizures^39^.

However, these alternative techniques will likely not replace vEEG and ECG as the “gold standard” for differential diagnosis, though they may be used as complementary screening and diagnostic tools. In short, a combined electroclinical analysis that utilizes clinical semiology, ECG and vEEG findings is effective for the differential diagnosis of epileptic seizures.

## 8. Treatment

**Figure 3:**
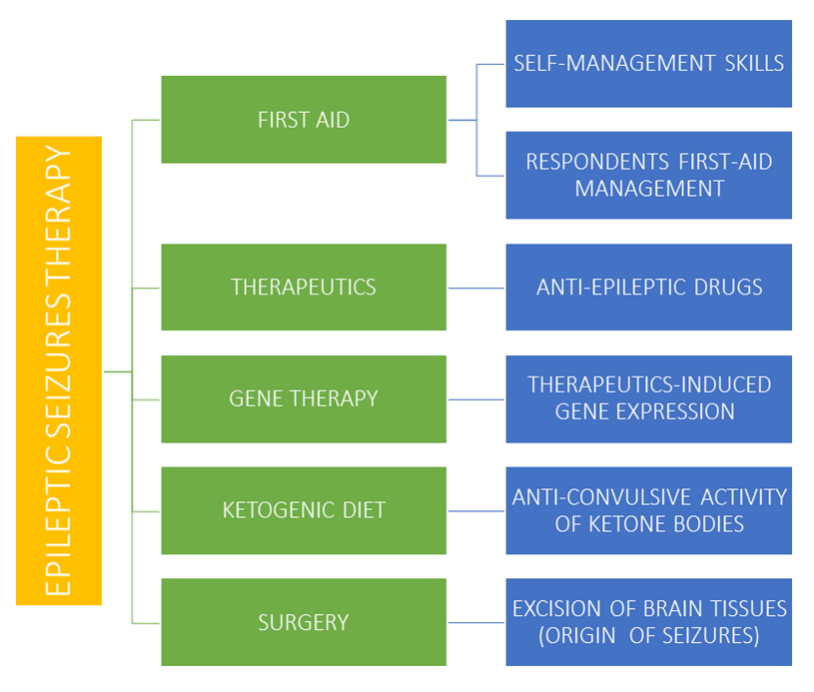
Treatment of epileptic seizures

The treatment for epileptic seizure includes first aid, therapeutics, gene therapy, ketogenic diet and surgery. These have been outlined in Figure 3**.**

### 8.1. First aid

First aid management refers to the action taken by people witnessing a patient who experiences an epileptic seizure. This includes helping others cope with the situation.

The negative attitudes and stigmas against people with epilepsy are more confusing than the disease. Misconceptions make people frightened when encountering an individual having an epileptic seizure. The actions of such witnesses play a key role. The most important task in this situation is to keep calm and help the patient. There are evidences that seizures can be safely managed by patients and their families if they have the correct training. Also, self-management intervention improves the confidence of patient and the situation is more effectively managed^41^. Self-management courses provided to patients at the epileptic centers help them reduce fear of seizure and improve their self-management skills^41^. The vital managing steps to be taken for handling an episode of seizure are shown in Table 2**.**

**Table 2:** First Aid management of epilepsy requires the following Do’s and Don'ts^41^

FIRST AID MANAGEMENT OF SEIZURES	Do’s and Don’ts
Run away from patient	NO
Clear area around the patient to avoid harm from object	YES
Let seizure run its course	YES
Forcibly hold the person down	NO
Put something soft under head of patient	YES
Restrain jerkiness	NO
Keep onlookers away from patient	YES
Monitor time of seizure	YES
Stand by the patient’s side whole time of seizure	YES
Maintain clear respiratory access	YES
Put something in patient’s mouth	NO
Roll patient onto their side	YES
Give antiepileptic medicine immediately	NO

It is important to know that seizure is not an emergency in which there is a need to call an ambulance immediately^42^. The Table 3 highlights the situations where there is dire need to call for rescue to take the patient to the hospital.

**Table 3:** Emergency situations when the ambulance needs to be requested^42^

A	B	C	D	E
				
				
				

After the duration of the seizure is monitored, the following must be checked: patients’ blood glucose level, pulse rate and respiration. Cyanosis may occur due to paralysis of respiratory muscles, but it must be made sure that it is temporary and normalizes when seizure subsides. The convulsive period of epileptic seizure rarely lasts longer than two to three minutes and rarely poses any life-threatening effects. Should, however, the seizure last for more than five minutes or if seizures occur with succession without the patient gaining consciousness in between, then it is an emergency situation and an ambulance must be immediately called. After the seizure ends, it is best to relax the patient and make him rest for some time. The patient will be sleeping for hours or even for a day after the seizure because of restlessness he faced during the attack^42^.

### 8.2. Drug-based therapeutics

The most important treatment option for epileptic seizures is thought to be the administration of the anti-epileptic drugs (AED) or anticonvulsive drugs. Many conventional AEDs are in use and newer ones are also undergoing experimental studies to determine their efficacy. About 65% of children have been known to be completely cured by AED if they are administrated at the initial stage of the disorder. There are many people who are AED-resistant and need alternative treatment. While taking the AEDs, special cautions must be taken to avoid interaction with drugs that can be harmful^43^. The AEDs must be taken only when prescribed by a doctor with a comprehensive look on the mechanism of interaction of the drug, its possible side effects and its proper dosage^43^. The tolerability, safety and efficacy of AEDs are important in determining their profile; these parameters help in practical use of AEDs against epileptic seizures. Although these drugs have different mechanisms of action to act against seizures, most of them are inhibitors which act on ion channels (like sodium or calcium channels) or neurotransmitters like GABA. Examples of AEDs include bumetanide, felbamate, ganxolone, regtibine, parampanel and carbamazepine^44^.Their mechanism, side effects and characteristics have been shown in Table 4**.**

**Table 4:** The AEDS under use; all mentioned are metabolized within the liver^44^

DRUG	MECHANISM	POSSIBLE SIDE EFFECTS	Characteristics
Bumetanide	Inhibits Na-K-Cl transporter	Dizziness, nausea, confusion	· Help in neonatal seizures · AED most effective in patients with temporal lobe epilepsy
Felbamate	Targets calcium channels and GABA	Agitation, behavioral changes	· US Food and Drug Administration (FDA) approved, good efficacy against seizures · Specific for epilepsy treatment
Ganaxolone	GABA modulatory activity	Dizziness and fatigue	· Effective for infantile spasm and adult partial seizures · Neurosteroid, mainly effective for partial onset of seizures
Retigabine	Activates potassium channels and GABA receptors	Aggressive behavior only in case of overdosage	· Safe and efficient · Unique mechanism of action
Perampanel	AMPA-glutamate receptor antagonist	Anger, irritability, aggression	· Epilepsy specific · Should not be given to hepatic disease patients · Broad spectrum and efficient AED
Carbamazepine	Shows anticonvulsive action when solubilized with chemically modified cyclodextrins	Confusion, agitation, aggression	· Intravenous administration is efficient against seizures · Poorly water soluble, mainly target sodium channels

New AEDs are being synthesized for seizures that are not efficiently treated with current ones. In some cases, newer AEDs are given in combination with conventional AEDs. These AEDs may be effective for drug resistant epilepsy (DRE). All of them, however, should not be used unprescribed. Some are already available like lamotrigine, vigabatrin, lacosamide, oxcarbazepine and some are in development^45^. These drugs are described in the Table 5**.**

**Table 5:** Characteristics of several anti-epileptic drugs^45^

Drug	Chemistry	Mechanism of action	Metabolism	Possible adverse effects	Characteristics
Topiramate	Sulfamate-substituted monosaccharide, broad spectrum AED	Blocks voltage dependent sodium ions. Inhibit GABA	70% excreted and 30% metabolized	Weight loss, cognitive problems, metabolic acidosis	Never withdraw drug promptly, but gradually
Vigabatrin	Structural analogue of GABA	Inhibits GABA transaminase	Renal	Insomnia, visual field defects, weight gain	Has been also used for infantile spasms
Stiripentol	AED used with other drugs, such as valproate, for the treatment of tonic-clonic seizures	Increases GABA release	Metabolized in liver	Minor central nervous system (CNS) effects	Not frequently prescribed
Zonisamide	Sulphonamide derivative that facilitate dopamine and serotonin transmission across blocked calcium channels	Acts on sodium channels and voltage dependent calcium channels	Metabolized in liver	Weight loss, CNS side effects, ataxia	Have drug interactions
Rufinamide	Triazole derivative, mechanism not exactly known	Prolongs inactive state of sodium channels	Metabolized in liver	CNS side effects	Not given to patients of familial short QT syndrome
Levetiracetam	Broad spectrum AED	Inhibits calcium channels, also lessens the calcium release from intraneuronal stores	Renal and enzymatic	Behavioral disturbances, headache, anorexia	Favorable safety profile, safe for liver disease patients
Lacosamide	Functionalized amino acid, AED used both oral and intravenous	Increases fraction of sodium channels for depolarization	Metabolized in liver	Nausea, Diplopia, CNS affected	Hypothesized to produce a neuroprotective effect
Lamotrigine	Broad spectrum AED, half-life affected by enzyme induced drugs	Inhibits voltage gated sodium channels	Metabolized in liver	Allergic reactions,	Can cause myoclonic seizures
Oxcarbazepine	10-keto analogue of carbamazepine	Inhibits voltage gated sodium channels	Metabolized in liver to active metabolite	Dizziness, CNS side effects, hyponatremia	Can make myoclonia worse

Most AEDs are metabolized in liver, whereas some in the kidney. There are certain factors that alter the action and effect of antiepileptic drugs as shown in Table 6**.**

**Table 6:** The factors that alter the action of AEDs^43^

Factors affecting the action of AEDs
Drug Dosage
Timing of dosage
Pharma Kinetics
Tolerance
Blood level
Interaction with other drugs

AEDs can have adverse effects on different systems of the body affecting function and efficiency. The most common side effects of AEDs include headache, ataxia, behavioural changes and allergic reactions. Several CNS functions are also affected^43^. The adverse effects are listed in Table 7**.**

**Table 7:** Side effects of AEDs^43^

Common adverse effects of AEDs intake
CNS disturbances
Behavioral changes
Cognitive effects
Weight changes
Psychiatric changes
Gastrointestinal Tract (GIT) disturbances

A patient manifesting epilepsy not responding to AEDs is said to have drug resistant epilepsy. The actual degree and pervasiveness of DRE and its mechanism is not yet clearly understood. Several hypotheses regarding DRE suggest that it is multifactorial, some are listed in the Table 8.

**Table 8:** Causes of DRE^43^

Causes of DRE
1. Intrinsic factors
2. Environmental factors
3. Seizures prior to diagnosis and treatment
4. Inception of seizures during neonatal life
5. Seizure-induced structural changes within the brain, making neuronal network abnormal

Comorbidity is seen in epileptic patients. This includes depression, suicidal thoughts, hepatic diseases, renal disease, osteoarthritis, attention deficit hypersensitive disorder, intellectual disability, psychiatric and behavioral comorbidities and neuropsychiatric disorders.

Several drugs, registered in the database of the USA National Institute of Health, are under clinical trials for epilepsy treatment. Several main examples are shown in Table 9 The type of study for all trials in the table is interventional.

**Table 9:** Clinical trials for epilepsy treatment registered in the database of the USA National Institute of Health (www.ClinicalTrials.gov)

Identifier	Study Design - Intervention Model	Study Design - Allocation	Study Design - Masking	Intervention	Phase	Status
NCT00212745	Single Group Assignment	Non-randomized	None	Behavioral: Andrews/Reiter behavioral treatment	I, II	Completed
NCT02983695	Single Group Assignment	N/A	None	Drug: TIL-TC150	I	Active, not recruiting
NCT02987114	Single Group Assignment	N/A	None	Drug: PLT101	II	Completed
NCT03370120	Single Group Assignment	N/A	None	Drug: padsevonil	II, III	Enrolling by invitation
NCT03428360	Single Group Assignment	N/A	None	Drug: DBSF (diazepam buccal soluble film)	III	Active, not recruiting
NCT01728077	Single Group Assignment	N/A	None	Drug: brivaracetam	III	Completed
NCT02726919	Single Group Assignment	N/A	None	Drug: clobazam	IV	Unknown
NCT01235403	Single Group Assignment	N/A	None	Drug: lacosamide	IV	Completed
NCT01689649	Single Group Assignment	N/A	None	Drug: topiramate	IV	Completed
NCT01311440	Parallel Assignment	Randomized	None	Other: modified Atkins diet treatment	N/A	Completed
NCT02076698	Parallel Assignment	Randomized	None	Procedure: anterior nucleus (of thalamus) deep brain stimulation (AN-DBS)	III	Active, not recruiting
NCT01405508	Parallel Assignment	Randomized	None	Drug: brivaracetam	III	Completed
NCT01645072	Parallel Assignment	Randomized	None	Other: low glycemic index diet	III	Unknown
NCT01028456	Parallel Assignment	Randomized	Double	Other: light therapy	N/A	Unknown
NCT00355082	Parallel Assignment	Randomized	Double	Drug: lamotrigine	III	Completed
NCT01745952	Crossover Assignment	Randomized	Triple	Device: Repetitive Transcranial Magnetic Stimulation (rTMS) coil	N/A	Completed
NCT03283371	Parallel Assignment	Randomized	Triple	Drug: natalizumab	II	Active, not recruiting
NCT01228747	Parallel Assignment	Randomized	Triple	Drug: levetiracetam	III	Completed
NCT01389596	Parallel Assignment	Randomized	Triple	Drug: pregabalin add-on therapy	III	Completed
NCT01999777	Parallel Assignment	Randomized	Triple	Drug: USL261	III	Completed
NCT03852303	Parallel Assignment	Randomized	Triple	Drug: ivermectin	IV	Completed
NCT03166215	Parallel Assignment	Randomized	Quadruple	Drug: TAK-935	I, II	Completed
NCT03796962	Parallel Assignment	Randomized	Quadruple	Drug: XEN1101	II	Recruiting

### 8.3. Surgery

Surgical intervention should only be considered when the subject does not respond to non-invasive therapies or medication. There is variation in surgical intervention, depending on the area of the brain where the seizure may occur or originate. Procedures include:

- Focal resection of the area causing the seizure is a procedure reserved for non-critical areas of the brain, as is the case with temporal lobe epilepsy (TLE), in medically intractable scenarios.
- Lesionectomy involves the removal of a well-formed brain tissue aberration (localized tumour or vascular deformation). Patients commonly present with glioneuronal tumours or meningiomas. Tumours have a high chance of being causative agents for epilepsy, and they present a good probability of seizure freedom with surgical intervention.
- A less invasive alternative may be found in laser interstitial thermal therapy (LIIT), which · depends on an MRI to map out the seizure focus and hence, guide a laser to eliminate that area of the brain only. LITT, as a treatment option for mesiotemporal epilepsy, shows a need for further improvement in parameters for surgery, because variations in anatomy between patients’ needs to be accounted for on a case-by-case basis.
- Corpus callosotomy involves severing the connection between both hemispheres and is reserved for severe generalized epilepsy (marked by tonic-clonic seizures and frequent seizure related falls). The procedure offers rather unreliable results, with only a fifth achieving seizure freedom post-surgery as a meta-analysis showed.
- Cases ill-suited for resection or ablation might be treated with the implantation of neurostimulation devices, but they confer palliative treatment as seizure freedom rates are reported as low. Regardless, the procedure has been determined to be safe and somewhat effective.

### 8.4. Ketogenic diet

Ketogenic diet (KD) is one which contains high fat content, low carbohydrates content and adequate protein content. This diet consists of high-fat in the form of long-chain triglycerides. As the fat metabolizes, it produces ketone bodies. The different ketone bodies, such as acetoacetate and beta hydroxybutyrate, are experimentally observed to have anticonvulsive role. Despite the new antiepileptic drugs, ketogenic diet is turning up to be a non-pharmacological substitute for treatment of epilepsy^51^.

The classic ketogenic diet is one that has calculated ratio of grams of fat to grams of carbohydrates plus protein. The most feasible ratios calculated until now are 3:1 or 4:1, with about 80-90% of the energy provided by fats and 10% by carbohydrates and proteins collectively^52^.

KD is most effective when taken after fasting or when the body's calorie levels are low. This is because the brain normally uses glucose as the source of energy, not fats. However, glucose levels are low in the fasting state, thus, the brain utilizes fats as the source of energy in this instance. Ketogenic diet has been used as a treatment for epileptic seizures due to its proven anticonvulsive role, and several research groups reported that KD therapy decreases the number of epileptic seizures in approximately 30-40% of children. Additionally, KD has also been seen to be effective for infantile epileptic seizures therapy^51^. It is mainly used as treatment in those patients who have DRE or for those patients who cannot undergo surgical excision procedure. Some guidelines concerning ketogenic therapy are shown in Table 10.

**Table 10:** Guide concerning ketogenic therapy^51^

Guide concerning Ketogenic Therapy	
Strict regular dietary requirements and medical supervision	Required
Limit for protein and fat consumption	Not required
Liquid restriction	Not required
Any possibility of side effects	Present

KD is not fully effective because it can cause several disorders, including but not limited to gastrointestinal disturbances and heart pathologies. Before initiating KD, the patient must be screened for disorders concerning fatty acid oxidation and metabolism^52^.

The exact mechanism of how a ketogenic diet performs anticonvulsive activity is still unclear. However, researches have shown that it decreases threshold level of a seizure. A KD affects both neurotransmitters and the neuronal membranes when acting as an anticonvulsant.

GABA is an inhibitory neurotransmitter widely distributed in neurons. Ketogenic diets have a role in synthesizing and maintaining high levels of GABA, which may be effective in treating epileptic seizures^51^. It is hypothesized that a KD decreases aspartate levels (induced by ketone bodies), which will facilitate the conversions of glutamate to glutamine and glutamine to GABA.

Ketogenic diet in neuronal membranes alters the vesicular glutamate transporter (VGLUTS). These are functioning by filling presynaptic vesicles and are chloride ion-dependent^1^_._ Ketone bodies, particularly acetoacetate, competitively inhibit chloride ion channels. The exact relation between VGLUTS and KD is still unknown. This ultimately leads to an increased in the inhibitory neurotransmitter GABA, and a decreased excitatory neurotransmitter glutamate.

KD therapies have shown efficacy for epilepsy. However, in order to use ketogenic diets for treatment, the strict protocols for usage must be followed as recommended by neurologists^52^. KD is in use for epilepsy and further clinical trials are being performed.**

### 8.5. Gene therapy

Many new antiepileptic drugs have been introduced in the past few years. However, almost one-third of epileptic patients still suffer seizures despite the use of AEDs^53^. Approximately 30% of people remain resistant to pharmacotherapy even with optimal treatment. Surgical removal of epileptogenic zone can prevent seizures, but it is unsuitable for more than 90% of patients with refractory epilepsy. Surgical intervention especially in case of focal neocortical epilepsy (FNE) has a lot of complications, as the functional areas of cortex can be damaged during the surgical procedure. Only a minority of the pharmacoresistant epilepsy patients can undergo a curative surgery. Gene therapy can be considered a treatment strategy for such intractable cases. This technique is very specific, since the therapeutic genes are introduced into the abnormal tissues only. In addition to the resistance of patients to AEDs, another problem observed is the development of several harmful side effects by the use of these drugs, which include mental retardation and lack of emotions, i.e. numbness. According to the FDA, some AEDs can even induce suicidal tendencies. Neurosurgery for the removal of the epileptogenic focus is the ultimate solution for patients with drug-refractory epilepsy, however, even then, the cure rate is not satisfactory. For such intractable cases, gene therapy can be considered as a treatment strategy^54^. Some genes, such as the neuropeptide Y and galanin, have shown a positive effect on the seizure activity. For the gene therapy treatment to be successful, the therapeutic gene must be accurately delivered to the target neurons.

The idea of gene therapy seems simple. Deoxyribonucleic acid (DNA) encoding therapeutic protein(s) is transferred via a vector into the abnormal cells in order to repair them permanently. Some genetic forms of epilepsy can be treated by transferring a healthy gene in place of the defective gene. Defective genes can also be repaired by the use of different gene editing technologies, including CRISPR (clustered regularly interspaced short palindromic repeats), Cas9-mediated genetic modification and CRISPRa (clustered regularly interspaced short palindromic repeats activation) or CRISPRi (clustered regularly interspaced short palindromic repeats inhibition). The transfer of genes capable of altering the function of the cell in relation to its hyperexcitability is another option which can be considered. There are several options available and several strategies could be adopted, but their practical application requires further investigation^55^.

Non-viral vectors have a lower tendency of provoking an immune response as compared to viral vectors. However, the major drawback of the non-viral vectors is low transduction efficiency. Experiments on different viruses for gene therapy have shown that adeno-associated viruses, lentiviruses, and herpes viruses are the most suitable for CNS application^56^. However, the complexity and diversity of the tissue being targeted and the presence of the BBB (Blood-Brain Barrier) pose hurdles for the *in vivo *human use of these viral vectors.

So far, the process of gene therapy for the treatment of epilepsy is done on animals through the focal application of vectors. Gene therapy has become a popular topic in the clinical world. However, its clinical application and practice in treating CNS dysfunctions faces many challenges. With rapid advancements of this field, it is not too far away the time until a successful gene therapy for epilepsy is performed^57^.

## 9. Conclusion

- Epilepsy is one of the most common neurological disorders affecting about fifty million people worldwide.
- There has been progress in classification of the epilepsy subtypes (based on the cause and commonalities) while etiology of acute epileptic conditions is still difficult to identify.
- While new tools and procedures have been added to aid diagnosis as well as differential diagnosis, the cornerstone of epilepsy related investigation remains vEEG combined with ECG. This possesses its own limitations, leaving a lot up to the skill of the clinician, history taking and witness testimonies.
- This article has revealed that the very nature of epilepsy is challenging to predict, since the patients only present to the clinicians upon suffering from a seizure or other relevant symptoms, yet headway is being made in the way of identifying biomarkers for the preictal period.
- Treatment with anticonvulsants is found to be well established, yet the refining of minimally invasive procedures for those with DRE remains to be desired.
- All in all, this review finds that leading a better life with epilepsy is feasible for most cases and can be improved with further breakthroughs in diagnostic and treatment procedures.
